# Obesity and Quality of Life in Young Women: Role of General and Central Adiposity

**DOI:** 10.7759/cureus.96740

**Published:** 2025-11-13

**Authors:** Carolina Piterschi, Lorina Vudu

**Affiliations:** 1 Department of Endocrinology, Nicolae Testemitanu State University of Medicine and Pharmacy, Chisinau, MDA

**Keywords:** body mass index, health-related quality of life, waist-to-height ratio, waist-to-hip ratio, young women living with obesity

## Abstract

Introduction: Obesity is a multifaceted disease and a growing public health concern that affects both physical health and psychosocial well-being. While its consequences are well documented in older adults, the impact of obesity on health-related quality of life (HRQoL) in young women remains insufficiently explored. Furthermore, limited evidence is available comparing how general and central adiposity differentially influence the various dimensions of HRQoL. Therefore, this study aimed to assess the impact of obesity on HRQoL in young women by comparing general adiposity and central adiposity across the physical, psychological, and social domains.

Methods: A cross-sectional study was conducted among 130 Caucasian women aged 18-45 years (mean age 31.8 ± 6.8 years); 69 with obesity (body mass index (BMI) ≥30 kg/m²) and 61 with normal weight (BMI 18.5-24.9 kg/m²). Anthropometric parameters were recorded, including BMI as an indicator of general adiposity and waist-to-height ratio (WHtR) and waist-to-hip ratio (WHR) as measures of central adiposity. HRQoL was assessed using the 36-Item Short Form Health Survey (SF-36), which evaluates eight domains and two composite scores: the Physical Component Summary (PCS) and Mental Component Summary (MCS).

Results: Women with obesity reported significantly lower HRQoL across all SF-36 domains compared with normal-weight participants (all p < 0.001). PCS and MCS scores declined progressively with increasing BMI, WHtR, and WHR, with the greatest impairments observed in class II and III obesity and in women with WHtR ≥0.60 or WHR ≥0.86. Correlation and regression analyses confirmed strong inverse associations between adiposity indicators and HRQoL, with central measures emerging as the most sensitive predictors.

Conclusions: Obesity in young women is associated with marked reductions in HRQoL across both physical and mental domains. The decline was most pronounced in women with class II and III obesity and elevated WHtR and WHR, reflecting the combined effects of general and central adiposity. These findings emphasize the need for early, multidisciplinary interventions targeting abdominal fat reduction and psychosocial well-being to enhance overall quality of life.

## Introduction

Obesity is a multifaceted disease and a growing public health challenge worldwide, affecting individuals across all age groups. According to data from the World Health Organization, in 2022, approximately 2.5 billion adults aged 18 and older were classified as overweight, including 890 million living with obesity [1]. Among adult women, the prevalence nearly tripled over the last four decades, rising from 6.6% to 18.5%, which corresponds to more than 500 million women affected globally [2].

The clinical burden of obesity is well established in older adults, where it contributes to chronic conditions such as type 2 diabetes, cardiovascular disease, osteoarthritis, chronic back pain, and other comorbidities [3,4]. Beyond its medical consequences, obesity markedly reduces quality of life by limiting physical functioning, impairing mobility, and contributing to emotional distress. Social stigma and weight-related discrimination further aggravate these effects, reinforcing a diminished sense of well-being [5-7]. Several studies have demonstrated that higher body mass index (BMI) is associated with a notable decline in quality of life, particularly among people living with obesity [8-14]. Large-scale analyses conducted in American populations have confirmed a continuous, inverse relationship between BMI and health-related quality of life (HRQoL), with physical and mental well-being declining progressively beyond a BMI of 20.4 kg/m². The decline appears steeper among women and younger adults, suggesting that adiposity may compromise quality of life early in adulthood [11]. However, most prior research has relied solely on BMI, while few have examined whether measures of central adiposity, such as waist-to-hip ratio (WHR) and waist-to-height ratio (WHtR), exert distinct effects on HRQoL, particularly in young women. Recent evidence indicates that WHR and WHtR show stronger associations with cardiometabolic risk and adverse health status. These indices better reflect central adiposity and visceral fat accumulation, which are metabolically active and more predictive of inflammation, cardiometabolic complications, and functional limitations such as fatigue, reduced mobility, and pain [15-17]. Psychosocial mechanisms may also play a critical role, as abdominal fat is more visible and stigmatized, particularly in young women, potentially exacerbating body image dissatisfaction, internalized weight stigma, and social withdrawal. Nevertheless, it remains unclear whether these parameters also influence psychological well-being and HRQoL in this population, which may be particularly vulnerable due to the early onset of metabolic alterations, societal pressures related to body image, and potential reproductive health implications.

Therefore, this study aimed to evaluate the impact of obesity on HRQoL in young women by comparing general adiposity (BMI) and central adiposity (WHR and WHtR) across the physical, psychological, and social domains, with the goal of generating evidence to guide targeted interventions. 

This article was previously presented as a meeting abstract at the European Society for Paediatric Endocrinology (ESPE) and the European Society of Endocrinology (ESE) in May 2025.

## Materials and methods

Study design and ethical approval

This cross-sectional study was conducted to evaluate the impact of obesity on HRQoL in young women. It included 130 Caucasian women aged 18-45 years who were recruited between November 2016 and December 2019 from the outpatient clinics and health promotion programs affiliated with the Department and Laboratory of Endocrinology, Nicolae Testemițanu State University of Medicine and Pharmacy, Chisinau, Republic of Moldova. Recruitment was conducted through voluntary participation following informational sessions and flyers distributed in university health centers. Eligible women were informed about the study, and those who expressed interest were screened according to the inclusion and exclusion criteria. All participants provided written informed consent prior to enrollment.

The minimum required sample size was calculated using standard formulas for comparing two proportions, with a significance level of 0.05 (Zα = 1.96) and a power of 90% (Zβ = 1.28). Based on the expected proportions of impaired HRQoL among women with normal weight (P0 = 0.27) and obesity (P1 = 0.58), the calculation indicated that at least 59 participants were required per group (approximately 118 in total). Ultimately, 130 women were enrolled, comprising 61 with normal weight and 69 with obesity, thereby exceeding the minimum sample size requirement.

Inclusion criteria were: (1) female sex, (2) age 18-45 years, (3) stable body weight (±2 kg) over the previous three months, and (4) absence of acute illness or chronic systemic disease. 

Exclusion criteria included: (1) overweight (BMI 25.0-29.9 kg/m²), (2) underweight (BMI <18.5 kg/m²), (3) pregnancy or breastfeeding, (4) menopause (natural, induced, or due to premature ovarian insufficiency), (5) obesity secondary to other diseases, (6) presence of comorbidities, or (7) current use of medication affecting metabolism or mood. 

To minimize sampling bias, all participants were screened and recruited by a single investigator according to the predefined eligibility criteria. Although the study focused on clinically healthy women to isolate the independent impact of adiposity on HRQoL, the voluntary nature of participation may have introduced self-selection bias, which is acknowledged as a study limitation.

The study protocol was approved by the Research Ethics Committee of Nicolae Testemitanu State University of Medicine and Pharmacy (Minute 4, November 4, 2016).

Anthropometric and clinical measurements

Anthropometric data, including body weight, height, and waist and hip circumferences, were obtained using standardized procedures with calibrated and validated equipment. Body weight was measured with a bioelectrical impedance analyzer (Tanita Body Composition Analyzer DC-360, Tokyo, Japan). Height was measured in centimeters (cm) using a stadiometer, from the soles of the feet to the vertex of the head. BMI was used as an indicator of general adiposity and was calculated as weight (kg) divided by height squared (m²), in accordance with World Health Organization recommendations. Participants were stratified according to BMI categories: normal weight (18.5-24.9 kg/m²), obesity class I (30.0-34.9 kg/m²), obesity class II (35.0-39.9 kg/m²), and obesity class III (≥40.0 kg/m²) [18]. Waist and hip circumferences were measured in the standing position at the horizontal plane using a flexible, non-stretchable plastic tape. Waist circumference was taken at the midpoint between the lower rib and the iliac crest, and hip circumference at the widest part of the buttocks. WHR and WHtR were subsequently calculated to assess fat distribution. Both indices served as measures of central adiposity, reflecting visceral fat accumulation. For WHR, values <0.80 were considered healthy, 0.80-0.85 indicated low to moderate risk, and >0.85 reflected an increased health risk in women. For WHtR, values <0.50 were considered healthy, 0.50-0.59 indicated increased risk, and ≥0.60 signified high risk [18,19]. 

All measurements were performed in the morning between 8:00 and 10:00 AM, following an overnight fast of at least eight to 12 hours. Participants were instructed to avoid vigorous physical activity, alcohol, and caffeine for at least 24 hours before assessment. Anthropometric evaluations were conducted in a quiet, temperature-controlled environment, with participants wearing light clothing and no shoes.

QoL assessment

HRQoL was assessed using the 36-Item Short Form Health Survey (SF-36, Version 1.0), a widely validated and reliable instrument recognized for its comprehensiveness, brevity, and alignment with current HRQoL assessment standards. The SF-36 evaluates perceived health status across multiple domains, encompassing both physical and mental components of well-being [20]. The SF-36 evaluates eight empirically distinct domains: physical functioning (PF) (10 items), vitality (VT) (four items), bodily pain (BP) (two items), general health perceptions (GH) (five items), physical role functioning (RP) (four items), emotional role functioning (RE) (three items), social role functioning (SF) (two items), and mental health (MH) (five items). Each domain was scored on a scale from 0 to 100, with higher scores indicating better perceived health status. In addition to the eight domain scores, two summary indices were calculated: the Physical Component Summary (PCS) and the Mental Component Summary (MCS). These were derived using weighted combinations of the eight domains according to standardized algorithms described in the SF-36 scoring manual. Both PCS and MCS are expressed on a scale from 0 to 100, where higher scores reflect better physical and mental health status, respectively. These summary scores provide a global view of the participants' physical and mental well-being. To ensure consistency throughout the manuscript, the terms “PCS” and “MCS” are used uniformly when referring to the composite SF-36 scores [21-24]. 

The SF-36 has demonstrated high internal consistency and test-retest reliability across diverse populations, including European and Eastern European cohorts, confirming its suitability for use in the Republic of Moldova. The instrument has also been shown to possess good construct validity for distinguishing between normal weight and obese groups in previous HRQoL studies [25,26]. In the present study, participants completed the SF-36 questionnaire in their native language under researcher supervision to ensure comprehension and completeness.

Statistical analysis

All statistical analyses were performed using the GNU PSPP software package, version 2.0.1 (Free Software Foundation (FSF), Boston, MA). Continuous variables were assessed for distributional characteristics using the Shapiro-Wilk test and visual inspection of histograms. Normally distributed data are presented as mean ± standard deviation (SD). Comparisons between the group with obesity (BMI ≥30 kg/m²) and the normal weight group (BMI ≤25 kg/m²) were analyzed using the one-way analysis of variance (ANOVA) procedure. Pearson correlation analysis was used to evaluate the linear relationship between HRQoL domain scores and anthropometric parameters. To examine independent associations, exploratory multiple linear regression analyses were subsequently performed, with HRQoL domain scores as dependent variables and BMI, WHR, or WHtR as predictors. Age was included as a covariate to account for developmental differences in perceived health status. Regression results are presented as unstandardized β coefficients, corresponding standard error (SE), and p-values. Regression assumptions were verified and examined by evaluating the normality, linearity, and homoscedasticity of residuals and by confirming the absence of multicollinearity (variance inflation factor < 5). A two-tailed p-value < 0.05 was considered statistically significant for all analyses.

## Results

A total of 130 Caucasian women (mean age 31.8 ± 6.8 years) were included, comprising 69 (53.1%) with obesity (BMI ≥30 kg/m²) and 61 (46.9%) with normal weight (BMI 18.5-24.9 kg/m²). The two groups were comparable in age (p = 0.665). Women with obesity had significantly higher body weight, BMI, WHR, and WHtR (all p < 0.001). Anthropometric and demographic data are presented in Table 1.

**Table 1:** Anthropometric characteristics of the study participants stratified by BMI Values are presented as mean ± SD. Statistical comparisons were performed using the ANOVA procedure. p < 0.05 was considered statistically significant. BMI: body mass index; WHR: waist-to-hip ratio; WHtR: waist-to-height ratio

Variables	Total sample	BMI ˂ 25 kg/m^2^	BMI ≥30 kg/m^2^	F value	p value
	n = 130 (100%)	n = 61 (46.9%)	n = 69 (53,1%)		
	(mean ± SD)	(mean ± SD)	(mean ± SD)		
Age, years	31.81 ± 6.75	32.08 ± 6.33	31.57 ± 7.14	0.19	= 0.665
BMI, kg/m^2^	28.96 ± 7.49	21.75 ± 1.93	35.3 ± 3.92	603.56	˂ 0.001
Waist circumference, cm	94.36 ± 19.69	75.75 ± 6.76	110.81 ± 10.50	497.60	˂ 0.001
Hip circumference, cm	107.98 ± 15.76	93.62 ± 4.36	120.67 ± 10.31	362.05	˂ 0.001
WHR	0.87 ± 0.08	0.81 ± 0.06	0.92 ± 0.05	137.97	˂ 0.001
WHtR	0.56 ± 0.12	0.45 ± 0.04	0.66 ± 0.06	542.37	˂ 0.001

Women with obesity reported significantly lower HRQoL scores across all eight SF-36 domains compared to women with normal weight (all p < 0.001). The largest impairments were observed in PF, MH, SF, and GH. Composite scores reflected these differences: the PCS decreased from 90.07 ± 5.34 in normal-weight women to 71.6 ± 13.97 in women with obesity, while the MCS decreased from 77.10 ± 6.14 to 59.31 ± 12.12, respectively (Table 2).

**Table 2:** SF-36 domain scores by BMI group Values are presented as mean ± SD. Statistical comparisons were performed using the ANOVA procedure. p < 0.05 was considered statistically significant. SF-36: Short Form-36; BMI: body mass index; PF: physical functioning; VT: vitality; BP: bodily pain; GH: general health perceptions; RP: physical role functioning; RE: emotional role functioning; SF: social role functioning; MH: mental health; PCS: Physical Component Summary; MCS: Mental Component Summary

Variables	Total sample	BMI ˂ 25 kg/m^2^	BMI ≥30 kg/m^2^	F value	p-value
	n = 130 (100%)	n = 61 (46.9%)	n = 69 (53,1%)		
	(mean ± SD)	(mean ± SD)	(mean ± SD)		
PCS	80.27 ± 14.2	90.07 ± 5.34	71.6 ± 13.97	94.36	˂ 0.001
PF	86.92 ± 16.38	96.89 ± 6.07	78.12 ± 17.58	62.89	˂ 0.001
RP	83.61 ± 12.26	89.14 ± 10.14	78.71 ± 11.95	28.37	˂ 0.001
BP	79.77 ± 20.0	91.68 ± 9.74	69.24 ± 20.86	59.17	˂ 0.001
GH	64.5 ± 18.42	76.56 ± 10.39	53.84 ± 17.41	78.96	˂ 0.001
MCS	67.66 ± 13.21	77.10 ± 6.14	59.31 ± 12.12	106.95	˂ 0.001
RE	80.96 ± 16.35	89.89 ± 9.99	73.07 ± 16.86	46.33	˂ 0.001
VT	57.69 ± 15.7	67.70 ± 6.99	48.84 ± 15.98	72.70	˂ 0.001
MH	63.45 ± 13.36	71.80 ± 7.28	56.06 ± 13.19	68.47	˂ 0.001
SF	78.17 ± 18.63	89.96 ± 11.6	67.75 ± 17.47	70.90	˂ 0.001

**Figure 1:**
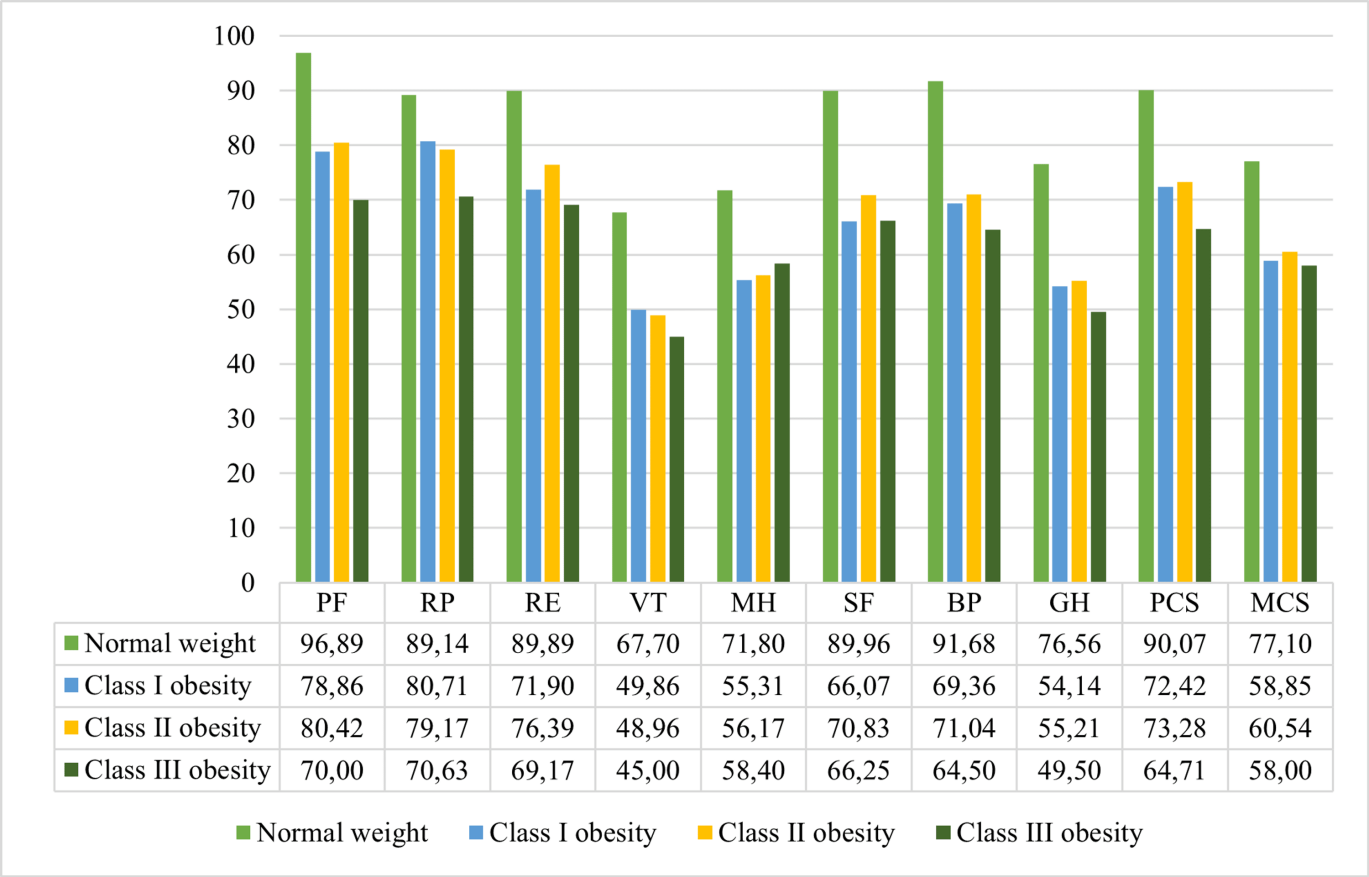
The mean SF-36 domain scores across BMI categories This figure illustrates the decline in health-related quality of life across the eight SF-36 domains with increasing BMI. SF-36: Short Form-36; PF: physical functioning; VT: vitality; BP: bodily pain; GH: general health perceptions; RP: physical role functioning; RE: emotional role functioning; SF: social role functioning; MH: mental health; PCS: Physical Component Summary; MCS: Mental Component Summary

When participants were classified according to BMI, 61 (46.9%) were normal-weight women, 35 (26.9%) with class I obesity, 24 (18.5%) with class II obesity, and 10 (7.7%) with class III obesity. HRQoL progressively declined across all domains (Figure 1). The steepest reductions were observed in PF and the PCS, highlighting the marked deterioration in physical health with increasing adiposity. Women with class III obesity reported the lowest VT (mean = 45.00) and GH (mean = 49.50), indicating substantial impairments in energy levels and overall well-being. Composite scores followed the same trend: PCS decreasing from 90.07 in normal-weight participants to 64.71 in class III obesity, and MCS decreasing from 77.10 to 58.00, respectively.

A similar graded association was found when HRQoL was analyzed according to WHtR and WHR categories (Table 3). Women with an optimal WHtR (˂0.5) and WHR (≤0.80) consistently reported the highest scores across all SF-36 domains. In contrast, those with WHtR ≥0.60 and WHR ≥0.86 had the lowest values, reflecting marked reductions in both PCS and MCS. For WHtR, the steepest impairments were evident in VT (68.09 ± 7.04 vs. 49.31 ± 16.42), MH (71.71 ± 7.05 vs. 56.34 ± 13.56), and GH (76.82 ± 10.69 vs. 54.48 ± 17.08). PCS scores declined from 90.01 ± 5.57 in the healthy WHtR group to 71.62 ± 14.28 in the high-risk group, while MCS scores decreased from 77.37 ± 5.95 to 59.92 ± 12.52. A similar pattern was noted for WHR: PCS dropped from 91.63 ± 3.83 in the ≤0.80 group to 74.33 ± 14.68 in the ≥0.86 group, and MCS from 78.93 ± 5.02 to 61.95 ± 12.82. Domain-level differences were particularly pronounced in PF (96.84 ± 4.09 vs. 81.01 ± 17.91), BP (93.00 ± 9.29 vs. 73.04 ± 21.33), VT (70.00 ± 6.16 vs. 51.33 ± 15.87), and MH (72.67 ± 6.81 vs. 58.78 ± 13.53).

**Table 3:** SF-36 scores according to WHtR and WHR categories *p ˂ 0.001 for all group comparisons. Values are presented as (mean ± SD). Statistical comparisons were performed using the ANOVA procedure. p < 0.05 was considered statistically significant. SF-36: Short Form-36; HRQOL: health-related quality of life; WHTR: waist-to-height ratio; WHR: waist-to-hip ratio; PCS: Physical Component Summary; PF: physical functioning; RP: physical role functioning; BP: bodily pain; GH: general health; MCS: mental Component Summary; RE: emotional role functioning; VT: vitality; MH: mental health; SF: social role functioning

HRQoL domains	WHtR			WHR		
							˂0.5	0.50–0.59	≥0.60	≤0.80	0.81–0.85	≥0.86
	n = 55 (42.3%)	n = 17 (13.1%)	n = 58 (44.6%)	n = 30 (23.0%)	n = 21 (16.2%)	n = 79 (60.8%)
	(mean ± SD)	(mean ± SD)	(mean ± SD)	(mean ± SD)	(mean ± SD)	(mean ± SD)
	PCS	90.01 ± 5.57	78.23 ± 13.92	71.62 ± 14.28	91.63 ± 3.83	86.38 ± 8.22	74.33 ± 14.68
PF	96.64 ± 6.32	86.47 ± 16.37	77.85 ± 17.87	96.84 ± 4.09	93.57 ± 9.64	81.01 ± 17.91
RP	89.43 ± 10.55	81.99 ± 9.60	78.56 ± 12.23	91.67 ± 9.89	87.2 ± 10.17	79.59 ± 11.87
BP	91.00 ± 9.91	78.53 ± 24.20	69.48 ± 20.43	93.00 ± 9.29	86.19 ± 13.93	73.04 ± 21.33
GH	76.82 ± 10.69	58.82 ± 19.81	54.48 ± 17.08	78.67 ± 8.90	71.43 ± 15.01	57.28 ± 18.20
MCS	77.37 ± 5.95	62.65 ± 12.68	59.92 ± 12.52	78.93 ± 5.02	73.04 ± 9.91	61.95 ± 12.82
RE	90.45 ± 9.94	74.02 ± 15.28	73.99 ± 17.18	93.06 ± 8.50	84.52 ± 14.02	75.42 ± 16.55
VT	68.09 ± 7.04	52.65 ± 14.37	49.31 ± 16.42	70.00 ± 6.16	64.05 ± 11.14	51.33 ± 15.87
MH	71.71 ± 7.05	60.94 ± 13.89	56.34 ± 13.56	72.67 ± 6.81	67.81 ± 11.69	58.78 ± 13.53
SF	90.45 ± 11.27	69.85 ± 15.35	68.97 ± 18.62	91.25 ± 9.93	86.90 ± 14.51	70.89 ± 18.53

These findings confirm that both general and central adiposity are strongly and inversely associated with HRQoL, with the most severe impairments observed in women with class III obesity, those with WHtR ≥0.60 or WHR≥0.86, underscoring their compounded impact on physical, mental, and social well-being.

The Pearson correlation analysis highlights that both the physical and mental dimensions of QoL are strongly influenced by the degree of adiposity, with more pronounced negative associations for BMI and the WHtR compared to the WHR. For the PCS, the strongest correlations were observed with BMI (r = -0.652) and WHtR (r = -0.624), followed by WHR (r = -0.494). Regarding the MCS, the negative correlations were also marked, especially for the WHtR (r = -0.633) and BMI (r = -0.617), followed by the WHR (r = -0.556) (all p ˂ 0.001) (Table 4).

In linear regression analysis, significant negative associations were observed between adiposity indicators and both physical and mental components of QoL (all p < 0.001). For the PCS, the strongest effects were linked to central obesity measures, with WHR (β = -91.45 ± 14.24) and WHtR (β = -76.27 ± 8.44), indicating that higher adiposity, particularly central, predicted lower PF, greater BP, and poorer GH. MCS showed a similar pattern, with WHR (β = -95.80 ± 12.66) and WHtR (β = -71.94 ± 7.78) showing the steepest declines, most evident in VT (β = -100.6 ± 15.79 for WHR) and SF (β = -119.38 ± 18.71 for WHR) (Table 4).

**Table 4:** Pearson correlations (r) and linear regression coefficients (β) (mean ± SE) between adiposity indicators and quality-of-life domains *p ˂ 0.001 for all group comparisons; p < 0.05 was considered statistically significant. HRQOL: health-related quality of life; WHTR: waist-to-height ratio; WHR: waist-to-hip ratio; PCS: Physical Component Summary; PF: physical functioning; RP: physical role functioning; BP: bodily pain; GH: general health; MCS: Mental Component Summary; RE: emotional role functioning; VT: vitality; MH: mental health; SF: social role functioning

HRQoL domains	Statistical associations	Anthropometric parameters		
		BMI	WHR	WHtR
PCS	Pearson r	-0.652	-0.494	-0.624
	Regression β ± SE	-1.24 ± 0.13	-91.45 ± 14.24	-76.27 ± 8.44
PF	Pearson r	-0.567	-0.416	-0.543
	Regression β ± SE	-1.24 ± 0.16	-88.82 ± 17.17	-76.52 ± 10.47
RP	Pearson r	-0.480	-0,413	-0.480
	Regression β ± SE	-0.79 ± 0.13	-65.98 ± 12.88	-50.61 ± 8.18
BP	Pearson r	-0.542	-0.430	-0.521
	Regression β ± SE	-1.45 ± 0.20	-65.98 ± 20.81	-89.65 ± 12.98
GH	Pearson r	-0.612	-0,453	-0.574
	Regression β ± SE	-1.51 ± 0.17	-108.83 ± 18.94	-90.96 ± 11.48
MCS	Pearson r	-0.617	-0.556	-0.633
	Regression β ± SE	-1.09 ± 0.12	-95.80 ± 12.66	-71.94 ± 7.78
RE	Pearson r	-0.477	-0.477	-0.508
	Regression β ± SE	-1.04 ± 0.17	-101.79 ± 16.56	-71.46 ± 10.71
VT	Pearson r	-0.583	-0.489	-0.585
	Regression β ± SE	-1.22 ± 0.15	-100.6 ± 15.79	-78.99 ± 9.69
MH	Pearson r	-0.517	-0.456	-0.533
	Regression β ± SE	-0.92 ± 0.14	-79.37 ± 13.71	-61.26 ± 8.60
SF	Pearson r	-0.542	-0.491	-0.532
	Regression β ± SE	-1.31 ± 0.19	-119.38 ± 18.71	-85.28 ± 12.01

These findings demonstrate that excess weight negatively impacts both physical and mental aspects of QoL, with WHtR emerging as the most sensitive and consistent predictor. This suggests that the accumulation of abdominal fat not only limits physical functioning but also substantially compromises emotional well-being and social participation, underscoring its critical role in the overall deterioration of QoL in women with obesity.

## Discussion

The present study examined the impact of obesity on HRQoL among young women, a demographic often underrepresented in obesity research. Despite being in a life stage typically associated with high physical functionality, social engagement, and productivity, women living with obesity reported significantly reduced HRQoL across all SF-36 domains compared to their normal-weight peers, with the largest impairments observed in vitality, general health, and physical functioning. These reductions were most pronounced in class II and III obesity and in women with elevated WHtR (≥0.60) or WHR (≥0.86), highlighting the compounded impact of both general and central adiposity.

Our findings are consistent with previous research demonstrating an inverse relationship between BMI and HRQoL. Rozjabek et al. (2020) reported a progressive decline in both PCS and MCS scores of the SF-36 with increasing general adiposity, with the steepest reductions between class II and class III obesity. They also found that higher BMI was associated with diminished patient activation and substantial impairments in work productivity and daily activity, underscoring the broader societal and functional consequences of obesity beyond health status alone [13]. Similarly, Jaison et al. (2024) highlighted strong associations between higher BMI and both physical symptoms (e.g., joint pain, fatigue) and psychological distress (e.g., irritability, anxiety), reinforcing our observation that obesity imposes a dual burden, physiological and psychological [27]. Tozetto et al. (2021), who examined the relationship between BMI, WHR, WHtR, and HRQoL, demonstrated that among young adults with obesity, WHtR was the anthropometric indicator most strongly associated with the physical component of quality of life. Importantly, no significant association was found between anthropometric measures and the MCS [14]. Our study was comparable to that of Tozetto et al., both in terms of sample size and mean age of participants (31.8 ± 6.8 years); however, in contrast to their findings, we observed impairments in both the physical and mental dimensions of QoL. A possible explanation for this discrepancy is that our study focused exclusively on women, a population that may experience a greater psychosocial burden of obesity due to body image concerns, social stigma, and gender-specific expectations. These factors may intensify the adverse impact of obesity on mental health and social functioning, thereby accounting for the broader decline in quality of life observed in our sample. 

Several interconnected mechanisms may underlie these findings. Physiologically, excess adiposity leads to systemic inflammation, insulin resistance, and biomechanical stress, which manifest as fatigue, pain, and functional limitations [3,4]. Psychosocial factors, including internalized stigma, body image concerns, and depressive symptoms, further compound the negative perception of health and social functioning [5-7,28]. This bidirectional interplay between physical and mental health reinforces a self-perpetuating cycle in which physical limitations intensify emotional distress, while poor mental health, characterized by diminished psychological well-being, emotional eating, physical inactivity, and stress-related hormonal dysregulation, reduces motivation for lifestyle modification and ultimately promotes weight gain and central fat accumulation [6,28].

Recognizing this multidimensional impact is essential for clinical practice and public health, underscoring the need for comprehensive management strategies that address both metabolic and psychological aspects of adiposity. If central adiposity carries disproportionate HRQoL impact, routine assessment should include waist-based indices alongside BMI to enhance risk stratification. Targeted waist-reduction strategies, combining physical activity interventions, nutrition optimization, and behavioral support, may yield improvements in both physical and psychosocial domains. Given the psychosocial pathways, integrating brief cognitive-behavioral strategies, body-image counselling, and stigma-informed communication into obesity care is warranted. At the population level, interventions that promote supportive environments for healthy behaviors and emphasize health and functional benefits over appearance may help reduce the psychosocial burden associated with abdominal obesity. Longitudinal and intervention studies are needed to determine whether reductions in WHtR or WHR translate into meaningful improvements in HRQoL and whether these effects differ across cultural contexts and psychosocial profiles.

Strengths of this study include the focus on young women, a population often underrepresented in obesity research, along with the use of a validated HRQoL instrument (SF-36), objective anthropometric measurements, and the incorporation of WHtR and WHR alongside BMI as complementary indicators of adiposity.

This study has several limitations that must be acknowledged. The cross-sectional design does not allow causal inference and does not exclude the possibility of reverse causation (i.e., impaired HRQoL contributing to weight gain and adiposity). Although standardized procedures were applied, minor measurement error remains possible, particularly in waist and hip circumference assessment. Recruitment through voluntary participation may have introduced self-selection bias, attracting women who are more health-conscious or motivated to engage in research. Additionally, the sample consisted exclusively of clinically healthy, Caucasian young women recruited from university-affiliated settings in Moldova, which may limit generalizability to other age groups, ethnicities, sociocultural contexts, or to men. Finally, the relatively small number of participants when subgrouped by BMI categories, especially those with class III obesity, may reduce the precision of estimates for these subgroups. 

Our findings underscore the importance of addressing both physical and psychological aspects of obesity in young women. Early identification of individuals at risk for impaired HRQoL, particularly women with elevated WHtR or severe obesity, may encourage timely, multidisciplinary interventions that improve overall well-being and help disrupt the cycle of obesity-related physical and emotional distress.

## Conclusions

This study demonstrates that obesity in young women is associated with reduced HRQoL, affecting both physical and mental well-being. Impairments were observed across all SF-36 domains, with the most pronounced reductions in PF, VT, and GH. Women with severe obesity and those with elevated central adiposity showed the greatest HRQoL deterioration, suggesting the multifactorial nature of obesity’s impact and underscoring the need for integrated interventions addressing both metabolic and psychosocial dimensions.

While these findings highlight the multidimensional burden of obesity in young women, the cross-sectional design limits causal inference. Future longitudinal studies should clarify temporal relationships between adiposity and HRQoL and explore potential mediating factors such as physical activity, mental health, and social stigma. Research including more diverse populations and intervention-based approaches is also needed to determine whether targeted lifestyle and psychosocial strategies can improve HRQoL outcomes in this vulnerable group.
